# Engineered Mesenchymal Stem Cells as Treatment for Cancers: Opportunities, Clinical Applications and Challenges

**DOI:** 10.21315/mjms2024.31.5.5

**Published:** 2024-10-08

**Authors:** Aishah Amirah Shamsul Kamal, Kamal Shaik Fakiruddin, Khadijat Abubakar Bobbo, King Hwa Ling, Sharmili Vidyadaran, Syahril Abdullah

**Affiliations:** 1 UPM-MAKNA Cancer Research Laboratory, Institute of Bioscience, Universiti Putra Malaysia, Selangor, Malaysia; 2 Haematology Unit, Cancer Research Centre, Institute for Medical Research, National Institutes of Health, Ministry of Health Malaysia, Selangor, Malaysia; 3 Department of Biomedical Sciences, Faculty of Medicine and Health Sciences, Universiti Putra Malaysia, Selangor, Malaysia; 4 Department of Pathology, Faculty of Medicine and Health Sciences, Universiti Putra Malaysia, Selangor, Malaysia; 5 Malaysian Research Institute on Ageing, Universiti Putra Malaysia, Selangor, Malaysia; 6 Malaysia Genome and Vaccine Institute, National Institutes of Biotechnology Malaysia, Selangor, Malaysia

**Keywords:** mesenchymal stem cells (MSCs), cell-based therapy, exosome-based therapy, tissue engineering, genetic modification

## Abstract

The insufficient and unspecific target of classical chemotherapies often leads to therapy resistance and cancer recurrence. Over the past decades, discoveries about mesenchymal stem cell (MSC) biology have provided new potential approaches to improve cancer therapy. Researchers have utilised the multipotent, regenerative and immunosuppressive qualities of MSCs and tropisms towards inflammatory, hypoxic and malignant sites in various therapeutic applications. Although MSC-based therapies have generally been demonstrated safe, their effectiveness remains limited when these cells are used alone. However, through genetic engineering, researchers have proven that MSCs can be modified to have specialised delivery roles to increase their therapeutic efficacy in cancer treatment. They can be made to overexpress therapeutic proteins through viral or non-viral genetic modification, which enhances their innate properties. Nevertheless, these engineering strategies must be optimised to increase therapeutic efficacy and targeting effectiveness while minimising any loss of MSC function. This review underscores the cutting-edge methods for engineering MSCs, discusses their promise and the difficulties in translating them into clinical settings, and offers some prospective suggestions for the future on achieving their full therapeutic potential.

## Introduction

Cancer is the most frequently diagnosed disease worldwide, accounting for more than 9 million deaths in 2020 (1). Third-generation compounds, such as vinorelbine and gemcitabine, paired with traditional chemotherapies and platinum drugs, like cisplatin or carboplatin, have been demonstrated to increase the overall survival rate of cancer patients. However, acquired chemoresistance and microscopic tumour spread would ultimately cause these treatments to fail (2).

Cancer stem cells (CSCs) are a rare subpopulation of cells within tumours that are known to promote tumour recurrence and chemoresistance (3). Nanoparticles (4–8) or antibody-conjugated nanoparticles (4, 5) have recently been used to target these CSCs. Although these approaches may appear promising, safety problems such as cellular toxicity and lack of specificity (6, 7) are some of the issues that may hinder their development toward clinical application. Due to the limitations of the current conventional therapies, the need to venture into alternative therapeutic strategies, such as cellular therapy, became necessary. For example, T cells genetically modified to express a receptor that identifies a specific antigen, known as the chimeric antigen receptor (CAR) T cells, have led to advancements in treating the tumour microenvironment (TME). CAR-T cells target the origin of tumours on the vascular side of inflammatory cytokines, where the TME forms and blocks immunosuppressive checkpoints. However, the efficacy of CAR-T cell treatment remains controversial because its toxicity damages organ structures, including neurological, pulmonary, cardiac and muscular structures, and causes fatal abnormalities (8).

**Figure 1:**
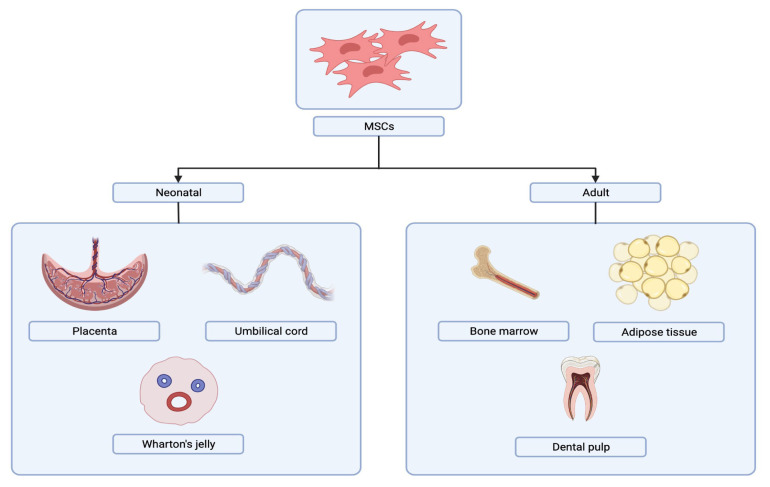
Sources of MSCs. MSCs can be obtained from various neonatal and adult tissues. Created in Biorender.com (11)

Another form of cellular therapy is the utilisation of mesenchymal stem cells (MSCs) as an initiative for targeted therapy. The International Society for Cellular Therapy (ISCT) specifies a set of basic criteria for defining human MSCs. First, MSCs must acquire plastic adherence when maintained under standard culture conditions. Second, MSCs must lack the expression of CD45, CD34, CD14 or CD11b, CD79 or CD19, and human leukocyte antigen-DR (HLA-DR) isotype surface molecules while expressing CD105, CD73 and CD90. Third, MSCs must have multi-lineage potential, enabling them to differentiate into adipocytes, chondrocytes and osteoblasts (9). Many sources of human MSCs (hMSCs) have been used in clinical settings. Figure 1 depicts that MSCs are derived from two sources. The first type is adult MSCs, which include bone marrow (BM), adipose-derived (AD) tissue and dental pulp, while the other is neonatal tissue-derived MSCs, obtained from the placenta, amniotic fluid and umbilical cord (10).

Currently, the experimental and clinical utilisation of MSCs encompasses many diseases and conditions, accompanied by potentially positive outcomes. MSC-based therapeutics has garnered vast interest and its application in cancer therapy continues to expand. However, the inability of transplanted MSCs to reach their maximum therapeutic potential is partially due to their lack of proliferative and differentiation ability associated with a lengthy culture period, inability to sufficiently migrate to the target site and inability to encounter hostility within the transplanted microenvironment, resulting in reduced engraftment time (12–15). These challenges led to the approach of engineering MSCs as an intervention.

Although various significant therapeutic findings have been linked to MSC-based therapy, there is always room for improvement to enhance the inherent properties and overcome the confronted challenges. The modification of MSCs has resulted in a highly specific and improved therapeutic approach in many experimental studies, while clinical trials are still in initial phases with preliminary objectives of assessing feasibility, efficacy and safety (16–18).

This review outlines an up-to-date report on preconditioning and genetic modification of MSCs as applied across several types of cancer. We discuss the application of MSCs in cancer, its limitations and why genetic engineering of MSCs is needed to enhance its antitumourigenic effects.

### Mesenchymal Stem Cells Features that Make Them Suitable for Cancer Therapy

#### Tumour Site-Specific Homing and Migration

Previous research has demonstrated that intravenously administered MSCs can home at specific tissues, encompassing tumour and injury sites. Migration of MSCs towards the tumour bed is activated by a signalling cascade similar to that in a chronic wound (19). Directional migration of MSCs towards the targeted tumour site is initiated in response to various poorly defined chemotactic stimuli released by inflamed tissues. Besides MSC-intrinsic factors, such as expression of migratory molecules, cell population heterogeneity and cell culture conditions, the tropism of MSCs towards cancerous sites is influenced by tumour site-intrinsic properties, such as inflammatory status, degree of vascularisation and oxygenation status (20).

Several types of molecules could influence the migration of MSCs to tumour sites. They include growth factors and their receptors, such as epidermal growth factor (EGF), fibroblast growth factor (FGF), hepatocyte growth factor (HGF), platelet-derived growth factor-AB (PDGF-AB), transforming growth factor β1 (TGF-β1), insulin-like growth factor 1 (IGF-1), vascular endothelial growth factor A (VEGF-A); chemokines, such as C-X-C motif chemokine ligand 12 (CXCL12), C-C motif chemokine ligand 2 (CCL2), CCL3 and their receptors (e.g. C-C motif chemokine receptor 4 [CCR4] or C-X-C motif chemokine receptor 4 [CXCR4]); cytokines, such as interleukin-6 (IL-6), IL-8 and tumour necrosis factor-alpha (TNF-α); and intercellular and vascular adhesion molecules (ICAM and VCAM) (21). These molecules are secreted by tumours and create a TME that mimics a wound-healing environment, enhancing the migration of MSCs to tumour sites.

The interaction between CXCR4 and stromal cell-derived factor 1 (SDF-1) was vital for the migration of MSCs within the BM (22). Interestingly, one major signal modulating MSC tropisms towards the TME is the binding of CXCR4 to macrophage migration inhibitory factor secreted by cancer cells. Previous in vivo research revealed that the downregulation of CXCR4 or macrophage migration inhibitory factor expression caused the migration of MSCs towards the pulmonary tumour metastatic site to be abrogated (23). There is evidence that the tumour tropism characteristics of MSCs are associated with additional receptors expressed by MSCs. For example, through paired CXCR4 and CXCR7 interaction with SDF-1, MSCs can get trapped in the lungs, rendering them incapable of migrating towards pulmonary cancer nodules (24). A study further confirmed the correlation that CXCR7 enhances MSC adhesion and migration toward osteosarcoma cells in vitro (25). Pathways such as CXCL16 binding with the CXCR6 receptor expressed by MSC can mediate cell docking into tumour sites (26). Tumour tropism characteristic of MSCs is influenced by several other vital signalling pathways, such as proteinase-activated matrix metalloproteinase-1 (MMP1) receptor 1 (27), urokinase-type plasminogen activator receptor (28, 29) and phosphoinositide 3-kinase (PI3K) (30).

#### Immunomodulation/Immunosuppression

It is generally accepted that allogeneic MSCs display low immunogenicity, allowing them to be transplanted to an allogeneic host without immunosuppression. The mechanism of action is based on their immunomodulatory properties and immunosuppressive activity. They can suppress the proliferation and activation of different immune system cells. These interactions may occur directly, such as in cell-cell interaction or indirectly via soluble factors.

The immunomodulating effect of MSCs is reflected in many T cell properties, such as activation and proliferation, and in this way, they efficiently suppress an immune response (31). The MSCs suppress the proliferation of activated T cells by secreting substances, such as indoleamine 2,3-dioxygenase (IDO) and prostaglandin E2 (PGE2) (32). They also suppress the development of pro-inflammatory Th17 cells and stimulate regulatory T cells by secreting immunosuppressive cytokines, including IL-6, IL-8, IL-10, TGF-β and HGF. In addition, the non-classical HLA class I molecules (HLA-G) expressed by MSCs exert immunosuppressive effects on various immune cells by inhibiting T cell proliferation and cytotoxic T lymphocyte-mediated cytolysis. Furthermore, they also induce the development of tolerogenic dendritic cells (DCs) and inhibit natural killer (NK) cell cytolytic functions (33, 34).

MSCs also regulate Th1/Th2 balance (T helper cells) by affecting the IL-4 and interferon-γ (IFN-γ) levels in effector T cells. MSCs disturb the maturation, differentiation and functions of DCs through cytokine secretion, which plays a crucial role in antigen presentation. There is also evidence that MSCs inhibit the proliferation, differentiation, and chemotaxis of B cells (35). Because of their immunoregulatory properties, MSCs are also protected against cell lysis and the cytotoxic effects of the host’s immune system.

The immunoprivileged and immunomodulatory nature of MSCs suggests that large quantities of these cells can be harvested from a healthy donor, expanded and manipulated ex vivo prior to infusion into multiple allogeneic patients as an ‘off-the-shelf’ therapy (36). The latter point not only renders this therapeutic strategy more practical in terms of time and cost but also alleviates ethical considerations related to re-infusing the cancer patient’s (autologous) cells about their potential to influence tumour malignancy.

#### Manipulation of Mesenchymal Stem Cells

Another impressive property of MSC is its ability to be genetically engineered (18), making it flexible as a drug carrier (37) while retaining its stemness characteristics. MSCs can be genetically manipulated to carry a range of molecules without changing their intrinsic properties. Hence, there has been a rise in MSC-based clinical trials, especially for cancer treatment (38). Several compounds have been tagged onto MSCs in vitro as a potential treatment for various disorders, including cancer. These compounds, which include chemicals, cytokines, hormones, proteins and vitamins, are introduced into MSCs via various methods (39). MSCs have been engineered with various antitumour molecules, including genes, oncolytic viruses, anticancer drugs and drug-encapsulated nanoparticles (40), as detailed in the next section. Importantly, MSCs have minimal side effects either with MSC-transfusion alone (41) or as an in-site drug delivery vehicle (42), exhibiting an extensive safety profile. Finally, the long-time culture of MSC for clinical applications such as transfusion showed little or no genomic alteration and genotoxicity (37).

### Enhancement of Mesenchymal Stem Cells for Cancer Therapy

Numerous in vitro and in vivo studies using MSCs in cancer therapy have been conducted. MSCs have been identified as a great tool for carrying and delivering several antitumour agents. The studies reported several modifications of MSCs for cancer treatment, among them viral and non-viral genetically engineered MSCs to deliver suicide genes such as TNF-related apoptosis-inducing ligand (TRAIL), which have shown strong antitumour activity in different types of cancers (43). Other genes can either knock down the expression (carrier for RNAi) or overexpress certain cytokines like the ILs or IFNs, especially IL-2, IL-12, IL-21, IFN-α, IFN-β or IFN-γ. Other modifications on MSCs are as carriers of established anticancer drugs, such as paclitaxel, gemcitabine or doxorubicin, or as a carrier for oncolytic viruses. Moreover, MSC-derived extracellular vehicles (EVs), such as the soluble TRAIL (sTRAIL) and exosomes, were explored recently. These therapeutic strategies were designed either as a stand-alone or combinatorial chemotherapy.

### Delivery of Cytokines (ILs and IFNs)

Proinflammatory cytokines employed in tumour immunotherapy play an important role at every stage of the cancer immunity cycle. They can repair antigen priming, increase the number of effector or immune cells in the TME, and improve their cytolytic abilities. Here, we analysed some of the most current studies on MSC-based cytokine delivery for cancer therapy.

#### Interleukins

In previous research, Nakamura and colleagues (44) discovered that MSCs exhibit migratory properties in tumour areas in 9-L glioma-carrying mice models via the corpus callosum, eventually invading the TME in vivo. Intratumoural injection of MSCs greatly slowed the growth of 9-L tumours and significantly extended the lifetime of 9-L glioma-bearing mice (44). Moreover, the results proved that injection of MSCs via an adenoviral vector expressing human IL-2 boosted anticancer effects and increased overall survival rates in tumour-bearing murine models, suggesting that MSCs can be used as cytokine delivery vehicles in anticancer therapy (44).

The upregulated expression of IL-2 in BM-MSCs slowed tumour progression in B16 melanoma-bearing mouse models. However, the parental BM-MSCs had no inhibitory effects on tumour formation in the transplanted models (45). Furthermore, injection of MSCs expressing IL-2 into B16 melanoma-bearing animals suppressed tumour progression by CD8 and NK cells but not by the activation of CD4 cells (45).

Furthermore, increased production of proinflammatory cytokines and enhanced peripheral blood mononuclear cells (PBMC) activation were observed in human IL-2 overexpressing adipose-derived MSCs (AD-MSCs) compared to parental stem cells. Conversely, the co-culture of human IL-2-producing AD-MSCs inhibited the viability of SH-SY5Y neuroblastoma cells in vitro (46). However, the finding suggested that AD-MSC-secreting IL-2-mediated therapeutic effects could be hampered by increased expression of pro-oncogenes, along with the natural potential of AD-MSCs to form malignancies (46).

Besides IL-2, injecting MSC-secreting IL-12 into animal models yields encouraging benefits. Previous studies show that subcutaneous, intratumoural and intravenous administration of IL-12-secreting MSCs elicited stronger anticancer properties and T-cell response with high tumour specificity compared to the injection of parental MSCs in murine models bearing B16F10 melanoma and TC-1 cervical tumour, respectively. However, the antitumour effect of intravenous and subcutaneous administration was significantly lower than intratumoural injection in both models. IL-12-secreting MSCs embedded in Matrigel (MSC-IL-12-Matrigel) also inhibited tumour growth in immunodeficient murine models, such as the severe combined immunodeficient murine (SCID) model, and beige/nude/X-linked immunodeficiency (BNX) mice lacking T and B lymphocytes (T and B cells) and NK cells, but not in IFN-γ knockout murine models (47).

Furthermore, the injection of MSCs engineered to overexpress IL-12 in B16F10 melanoma-bearing mice models significantly reduced melanoma lung metastases by inducing the activation and proliferation of NK cells (48). Moreover, significant inhibition of tumour progression and enhanced survival rate was observed following subcutaneous administration of IL-12-secreting MSCs into tumour-bearing mice (48). Due to the ability to home to tumours and generate local IL-12, systemic injection of BM-MSCs transduced with a recombinant adenoviral vector expressing murine IL-12 in mice models bearing renal cell carcinoma (RCC) slowed tumour growth and markedly prolonged mouse survival (49). According to the findings, the antitumour effects in animal models were linked to the presence of IFN-γ and NK cells (49).

Aside from the evidence exhibiting the therapeutic potency of IL-12-secreting BM-MSCs in Ewing sarcoma tumours (50), another study indicated their anticancer effects in murine metastatic hepatoma established by HCA-I and Hepa 1–6 cells. The underlying mechanisms for the modified MSC-exerted anticancer effects were described as an upregulated expression of monocyte chemoattractant protein-1 (MCP-1/CCL2) and enhanced stimulation of NK cells and cytotoxic T lymphocytes (CTL) in tumour tissues (51). Furthermore, treatment suppressed pulmonary metastasis and improved survival rates in tumour-bearing mice by inducing apoptotic activity in tumour cells by activating the proliferation of effector immune cells (51).

Current research suggests that utilising MSCs to deliver IL-21 could exert anticancer effects in many malignancies by regulating immune cell proliferation and activation. For example, systemic injection of umbilical cord-derived MSCs (UC-MSCs) secreting IL-21 into A2780 ovarian cancer-bearing mice models increased cytotoxicity towards NK cells and the number of IFN-γ-producing splenocytes in transplanted models (52). On the contrary, the intervention improved the survival rates of tumour-bearing mice and inhibited tumour development (52).

Similarly, the administration of human UC-MSCs secreting IL-21 into SKOV3 nude mice with ovarian tumours reduced tumour burden in the transplanted mice, as seen by the decreased tumour size (53). It is noteworthy that the delivery of IL-21 by human UC-MSCs altered the expression of TNF-α and IFN-γ in the murine serum, increased the expression of major histocompatibility (MHC) class I polypeptide-related sequence A (MICA) and NK group 2D (NKG2D) molecules in tumour tissues, and finally reduced the expression of cyclin-D1 and β-catenin in the tumour, resulting in hampered tumour growth post-transplantation (53). Local administration of MSCs secreting IL-21 into B-cell lymphoma BALB/c murine models was reported to prolong anticancer effects, inhibiting the formation of tumour nodules (54).

Nevertheless, although the activation of NK and effector T cells by IL-21-MSC treatment reduced tumour progression and improved survival in transplanted mice, no significant anticancer responses were observed following the injection of MSCs with recombinant adenovirus-expressing IL-21 (rAD/IL-21) in the mice (55).

#### Interferons

MSCs, according to previous research, have a high potential to deliver IFNs to tumour tissues to induce immune cell antitumour characteristics (56). A previous study discovered that IFN-α-overexpressing BM-MSCs may inhibit lung metastasis in B16F10-bearing C57BL/6 mice with metastatic melanoma. As a result, in experimental models, administration of IFN-producing MSCs via systemic infusion slowed tumour progression and prolonged survival. Immunohistochemistry evaluation revealed increased apoptotic activity and decreased proliferation and blood vasculature, indicating that adult IFN-overexpressing MSCs can slow the progression of melanoma lung metastasis (57). Previous research also revealed that even a relatively small population of MSCs capable of producing IFN-α may significantly slow the progression of B16 tumours in xenograft models due to the activated CD8-positive T cells and NK cells (58).

Researchers have also incorporated different IFNs with MSCs. Yang et al. (59) hypothesised that MSCs that continuously produce IFN-γ could kill tumour cells by continuing to activate the TRAIL pathway, which induces apoptosis. In turn, IFN-γ-modified MSCs could produce a large amount of functional IFN-γ, resulting in the prolonged production secretion of TRAIL and subsequent activation of the caspase cascade in tumour cells. For instance, after improving TRAIL expression in tumour cells in vitro, IFN-γ-secreting MSCs selectively activated the apoptotic pathway in lung tumour cells by upregulating caspase-3 activation. Furthermore, in xenograft murine models, MSCs producing IFN-γ slowed the progression of lung carcinoma (59).

In a previous study, murine macrophages were polarised to the M1 phenotype by MSCs producing IFN-γ in vitro, and these IFN-γ-secreting MSCs additionally inhibited tumour growth in neuroblastoma tumour cell-bearing xenografts, resulting in ameliorated overall survival (60). Furthermore, co-culturing IFN-γ-overexpressing BM-MSCs with human chronic myelogenous leukaemia (CML) K562 cells resulted in strong inhibition of leukemic cell apoptosis and cell proliferation, along with the induction of G1 phase cell cycle arrest (61).

MSC-producing IFN-β exhibited a more efficient antitumour impact than IFN-α and IFN-γ gene delivery by MSCs. Intravenously injected MSC can migrate to the breast tumour region and secrete high amounts of IFN-β into the tumour stroma, according to previous research on breast tumour xenografts (62). Meanwhile, intratumorally generated IFN-β was found to suppress primary tumour growth and attenuate pulmonary and hepatic metastases by downregulating activation of matrix metalloproteinase-2 (MMP-2), signal transducer activator transcription factor 3 (Stat3), cellular Myc (c-Myc), protein kinase B (Akt), and proto-oncogene tyrosine-protein kinase **(**Src) expression in tumour cells (62).

Furthermore, evaluation of the antitumour effects of combined therapy with canine AD-MSCs producing IFN-β and cisplatin in B16F10 melanoma-bearing mice revealed that intratumoural administration of cisplatin combined with subcutaneous injection of engineered AD-MSCs exhibited superior effects compared to the administration of modified AD-MSCs or cisplatin alone in terms of melanoma tumour development inhibition and survival rate (63). Moreover, positive results from the treatment of human U87 glioma-bearing murine models with MSCs secreting IFN-β indicated that MSCs can migrate into brain tissue following local or systemic delivery, allowing for the treatment of human glioma (64). Furthermore, injection of IFN-overexpressing MSCs resulted in a remarkable decrease in tumour volume in a xenograft model of prostate cancer lung metastasis (57) and a xenograft model of squamous cell carcinoma (65).

### Suicide Gene

Several studies have used viral vectors for directed enzyme prodrug therapy (GDEPT) or suicide gene therapy. MSC-targeted suicide gene therapy is a two-step process. Transduction of MSCs targets the gene for the foreign enzyme (bacterial, yeast or viral) to the tumour in the first step. Transcription of the gene encoding the prodrug-drug converting enzyme will produce a deadly substance at the tumour site in the second step. According to earlier research, the retroviral suicide gene construct is frequently employed for MSC transduction, with CD and HSV-TK being the most transduced suicide genes. AD-MSCs and BM-MSCs have been used as delivery vectors for anti-neoplastic drugs (66). In terms of proliferation, differentiation, tumour-homing potentials and surface antigenicity, modified MSCs exhibited no differences apart from naïve MSCs (67).

MSCs, like most healthy tissues, exhibit very low amounts of receptors and are unaffected by TRAIL-induced apoptosis. Hence, recruiting MSCs that secrete TRAIL leads to excellent cancer treatment outcomes (68). Furthermore, co-transfection of the TRAIL gene with various double suicide gene techniques (e.g. HSV-TK) that face challenges such as antagonistic antitumor activity could boost the tumouricidal effect efficacy (69). The median volume of injected therapeutic cells is suggested to be less than 10% of the tumour mass to promote efficient MSC homing to the tumour location (70).

Subsequently, it is practical to employ multiple low-dose injections of therapeutic cells rather than a single high-dose injection. Kim et al. (71) demonstrated that mice with metastatic RCC exhibited a 50% and 100% survival rate following two and three small-divided doses of MSC/TRAIL-TK injections. Another study looked at the effectiveness of consecutive suicide gene therapy, verifying that repeated administrations of Cdy: UPRT-AD-MSC into the cerebrum resulted in an 88% increase in the survival period of rats with glioblastoma (GBM) (72).

Some studies used combined therapeutic approaches for the treatment of malignant disease. In some circumstances, combining suicide gene-MSCs with chemotherapeutic drugs produces satisfactory results. Ando et al. (73) discovered that when the proteasome inhibitor bortezomib was combined with MSC-Ad.iC9, non-small cell lung cancer (NSCLC) development was prevented. When administered on its own, the medication is unsuccessful in treating NSCLC. Meanwhile, another investigation used MSCs-TK and valproic acid (VPA) to treat glioma-bearing mice (74). In the murine model, the combined treatment significantly inhibited tumour growth and increased the survival rate.

### RNA Interference

MSCs have piqued the interest of researchers and exhibited good potential as a cellular treatment and RNAi targets. RNAi has been employed to ameliorate the therapeutic benefits of MSCs in many disorders and one such strategy is RNAi-based functional modification (75). MicroRNA (miRNA) regulates stem cell migration and homing capabilities (76) and its alteration could improve the efficiency of MSC-based therapy. RNAi-based stem cell modification could effectively control restrictions to stem cell application, such as stem cell-related fibrosis, which is generated by spontaneous fibroblastic differentiation of stem cells (77, 78). The secretion of specific cellular factors could be induced by RNAi-mediated gene silencing. Suppression of miR-383 can increase glial cell line-derived neurotrophic factor (GDNF) production, which could assist human bone marrow-derived MSCs to repair spinal cord damage more effectively (79).

### Oncolytic Viruses

As a novel cancer treatment technique, virotherapy has various advantages, including the possible lack of cross-resistance with conventional therapies and the ability to promote tumour elimination through various mechanisms. The self-preserving characteristic of oncolytic viruses (OVs) acts as a superior approach for inserting the therapeutic transgene, regardless of the unique properties that distinguish them from other treatment options. The utilisation of MSCs as a non-systemic carrier of OVs for treating many clinical diseases has been studied successfully. The powerful anticancer effects of the OVs delivered by MSCs into tumour tissues are discussed in this section.

#### Oncolytic Adenovirus

In the research investigating the immune reaction to oncolytic adenovirus-transduced MSCs (MSC-oAd) in semi permissive cotton rat (CR) model, Ahmed and colleagues (80) discovered that CR-MSCs can maintain the replication of oncolytic adenovirus (oAd) in vitro. Moreover, CR-MSCs can limit IFN-γ production by activated T cells while promoting the distribution and persistence of oAd compared to viral injection alone in vivo. This suggests that utilising MSCs as a delivery method for oAd could have multiple benefits, including supported delivery, increased distribution, and reduced virus persistence by inhibiting antiviral immune responses (80).

Furthermore, intravenous injection of MSC-oAd was demonstrated to home in xenograft hepatocellular carcinoma (HCC) tumours, inducing virion accumulation in the tumours, subsequently resulting in a significant tumour growth inhibition (81). In both hypoxic and normoxic conditions, MSC-oAd can induce apoptosis in HCC cells in vitro, implying that MSC-linked systemic oAd transport is a potential means of achieving synergistic antitumor efficacy with enhanced safety profiles (81). Similarly, MSC-oAd exhibited antitumor properties in orthotopic mouse models of breast and lung tumours after systemic injection, resulting in enhanced survival of xenograft models, whereas only transduction of the liver was observed after the administration of oAd without MSCs as a vector (82).

When human MSCs transduced with conditionally replicating adenoviruses (CRAds) were systemically administered into SCID murine xenograft metastases model of breast tumours, enhanced migration of injected cells into tumour site was observed in in vivo models, ameliorating murine survival rates compared to murine models treated with only CRAds, where this observation is suggested to be linked to MSCs facilitating viral amplification (83). On the contrary, Guo and his colleagues (84) discovered that MSCs derived from menstrual blood (Men) and loaded with oAd could have strong inhibitory effects on the progression of colorectal cancer (CRC) tumours in mice models, possibly due to viral amplification, as exhibited by higher amounts of viruses accumulating in the TME. Besides exhibiting inhibitory properties on prostate cancer cell proliferation in vitro, MSC-oAd displayed tumour-homing characteristics in prostate cancer mouse models, accompanied by tumour growth suppression in experimental models. This result was consequentially obtained from the consistent replication of the virus, allowing the commencement of cell lysis (85).

#### Oncolytic Herpes Simplex Virus

MSCs transduced with oncolytic herpes simplex virus (oHSV, MSC-oHSV) can develop oHSV progeny capable of lysis of GBM cells in vitro and in vivo, potentially through a dynamic oHSV infection and tumour eradication pathway (86). Furthermore, biocompatible synthetic extracellular matrix (sECM)-encapsulated MSC-oHSV can induce enhanced anti-GBM potency compared to direct utilisation of isolated oHSV in the xenograft model, resulting in an extended lifespan in animal models (86). Furthermore, MSC loaded with oHSV-TRAIL can trigger apoptosis-related death and prolong survival in mice with oHSV-TRAIL-resistant GBM (86).

Likewise, systemic administration of MSCs infected with a HER2-retargeted oHSV led to the propagation of the oHSV progeny from MSCs to tumour cells in ovarian cancer and metastatic breast murine models (87). The presence of a significant concentration of MSCs and viral genomes in the lungs of mice was confirmed through observation, as was the reduced tumour development in nude mice (87).

MSCs infected with HF10, an HSV-1 mutant, exhibited antitumour properties in vitro when combined with the tyrosine kinase inhibitor erlotinib, revealing that combination therapy exerted significant cytotoxic activity toward human pancreatic cell line BxPC-3. However, no significant cytotoxicity was observed against human pancreatic cell line PANC-1 cells (88). In the subcutaneous tumour model, the combination of MSC-HF10 and erlotinib supported sustained viral proliferation in tumour regions and more significant suppression of tumour progression than utilising either alone (88).

#### Oncolytic Measles Virus

Human MSCs infected with oncolytic measles virus (oMV, MSC-oMV) have already been shown to produce targeted therapeutic effects in a range of human cancers, such as ovarian cancer (89). In this regard, investigations in SKOV3ip.1 ovarian tumour xenografts exhibited that MSCs administered intraperitoneally may migrate directionally towards peritoneal tumours, home to the tumour parenchyma, subsequently transmitting virus infection to tumours in both measles-naive and passively immunised murine models. Surprisingly, the MSC-oMV, but not an uninfected MSC or a naked virus, increased the survival rates of orthotopic ovarian cancer models upon administration (89). Furthermore, Mader and colleagues (90) also discovered that patient-derived MSC can be pre-infected with oMV, frozen in liquid nitrogen and thawed prior to being administered without the risk of efficacy deterioration.

In addition, when BM-MSC-oMV is systemically administered into human HCC SCID murine models, significant tumour growth inhibition was observed in both measles antibody-naive and passively immunised SCID animals. Nevertheless, administration of MV viruses alone induced antitumour effects only in measles antibody-naive SCID mice, indicating that MSC-oMV could be utilised to treat human liver cancers (91). MV transport via MSCs holds promise for treating haematological cancers despite the positive results seen in solid tumours. As a result, systemic injection of the BM-MSC-oMV in xenograft models of acute lymphocytic leukaemia (92) and multiple myeloma (93) induced antitumor responses, such as suppression of cancer progression through induced apoptosis of malignant cells, contributing to enhanced survival in transplanted models.

### Anti-Angiogenic Agents

Angiogenesis is a key process in the development and progression of gliomas. One of the glioma therapeutic approaches is thought to involve agents that suppress angiogenesis. Pigment epithelial-derived factor (PEDF) is a secreted glycoprotein with a molecular weight of 50 kDa, which can initiate the activation of the Fas/FasL pathway to trigger endothelial apoptosis and maintain the balance between angiogenesis inducers and inhibitors (94, 95). Zhang et al. (96) reported that PEDF was involved in the angiogenesis and tumourigenesis of gliomas. Meanwhile, Wang et al. (95) demonstrated in 2013 that MSCs expressing PEDF successfully triggered tumour cell death and blocked angiogenesis, shrinking the volume of the tumour site and increasing the survival rate of glioma-bearing murine models. However, the molecular mechanism of PEDF triggers anti-angiogenic activity and apoptosis in glioma is still unknown. Engineered MSCs operate as an inhibitory molecular carrier in gliomas, promoting apoptosis and inhibiting angiogenesis, and may have therapeutic potential stem cell treatment against gliomas in clinical settings.

### Pro-Apoptotic Ligands and Proteins (TRAIL)

**Figure 2:**
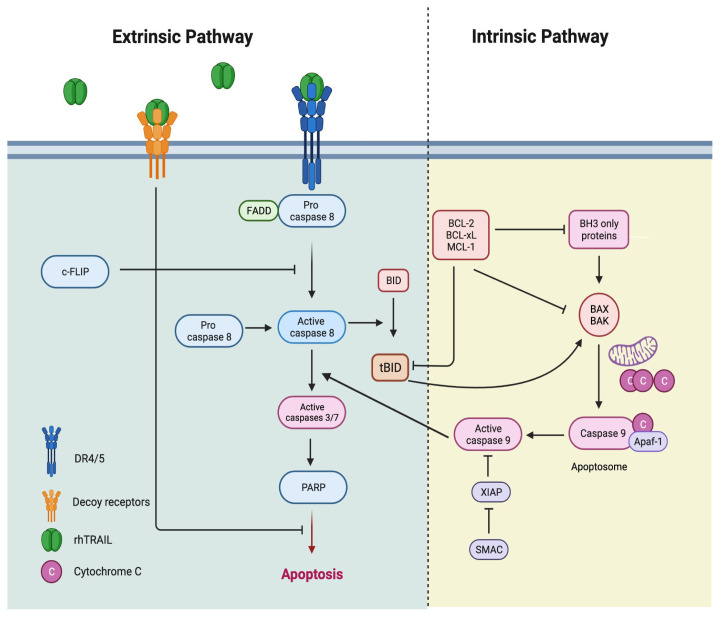
The TRAIL-induced apoptosis pathway and its regulation. Activation of DR4 and DR5 by TRAIL induces the extrinsic apoptosis pathway (left). The intrinsic pathway (right) is activated by various stimuli, rendering the secretion of proapoptotic proteins from the mitochondria. The interaction between the two pathways is initiated when caspase-8 activated by the DRs can cleave BID, which then activates the intrinsic pathway. Contrariwise, caspase-3 can cleave and activate caspase-8 in a feedback loop, amplifying the apoptotic signal. Decoy receptors do not induce apoptotic pathways due to the absence of functional death domains. Created in Biorender.com (97)

Despite the possibility of adverse consequences, apoptosis is an effective mechanism for tumour therapy. TRAIL, otherwise known as Apo-2 ligand (Apo2L), is an ideal anticancer cytokine due to its unique ability to target specific targets and exert its effect on cancer cells while sparing normal cells. Figure 2 illustrates that TRAIL binds with two receptors, TRAIL-R1 (alternatively known as DR4) and TRAIL-R2 (alternatively known as TRICK2, KILLER, Apo2, and DR5), to induce the formation of a death-inducing signalling complex (DISC). This complex comprises a trimerised receptor and the adaptor protein Fas-associated protein with death domain (FADD). FADD recruits procaspase-8 to DISC, which activates caspase-8 by auto-catalytic cleavage and formation of homodimers. Upon release from DISC, activated caspase-8 cleaves and activates caspase-3, the ‘effector’ of the extrinsic apoptosis pathway. Further on, an intrinsic apoptotic pathway is activated by TRAIL through the cleaving of BH3 interacting-domain death agonist (BID) into truncated BID (tBID), which translocates to the mitochondria, rendering the release of Smac/DIABLO and cytochrome c. Apaf-1 and cytochrome c activate caspase-9, which subsequently activates caspase-3 (97). In contrast to TRAIL, most traditional chemotherapeutics and radiotherapeutics require the activation of p53 to induce intrinsic apoptosis pathways (98). Therefore, TRAIL is superior to conventional cancer therapy to induce apoptosis in tumours with p53 deletions or mutations.

In addition to TRAIL-R1 and TRAIL-R2, TRAIL can also bind to two non-DD-containing membrane receptors called TRAIL-R3 (decoy receptor 1, DcR1) and TRAIL-R4 (decoy receptor 2, DcR2) (99, 100). TRAIL-R3 is a glycosylphosphatidylinositol-anchored receptor without an intracellular domain, and TRAIL-R4 contains a truncated, non-functional DD in its intracellular domain. Compared to most transformed cell lines, high expression of TRAIL-R3 is observed in normal cells, such as the placenta, BM, kidney, liver, heart, lung, spleen, and peripheral blood lymphocytes (101). The extracellular domains of TRAIL-R3 and TRAIL-R4 do not possess the capability to induce the apoptotic pathway despite their similar characteristics to the extracellular domains of TRAIL-R1 and TRAIL-R2. When TRAIL-R3 and TRAIL-R4 are highly expressed, these decoy receptors with non-functional or truncated DD will compete with the death receptors (DRs) TRAIL-R1 and TRAIL-R2 harbouring the functional DD, leading to the inhibition of the TRAIL-induced apoptotic pathway (102, 103).

As mentioned, TRAIL receptors, known as DRs, are predominantly expressed in cancer cells (100, 104). In this context, both the wild full-length membrane-bound protein (FL-TRAIL) or a modified or recombinant soluble TRAIL (sTRAIL) have been explored, with remarkable preclinical antitumor results (105, 106). Homing, short half-life and reduced bioavailability were the main clinical limitations of sTRAIL, hence the option of an MSC as the delivery vehicle. Tracking and biodistribution of 89Zr-oxine labelled MSC-TRAIL with PET/CT imaging in metastatic lung adenocarcinoma xenograft models demonstrated effective delivery of MSC-TRAIL up to one-week post-injection (105).

Compared to cells expressing FL-TRAIL, MSC-sTRAIL can significantly induce more potent apoptosis-inducing activity. Although TRAIL can stimulate the expression of prometastatic molecules like CXCL5/ENA-78 and IL-6 in prostate cancer cells, this effect can be countered using an AKT inhibitor combined with TRAIL (107). As a result, combining MSC-sTRAIL with small-molecule drugs could sensitise tumour cells to TRAIL while reducing the risk of cytokine secretion, which can cause side effects (107). In contrast, another study found that MSC-FL-TRAIL may cause higher cytotoxicity against cancer cells than MSC-sTRAIL, as well as resistance of cancer cells towards recombinant TRAIL, implying that MSC-FL-TRAIL is superior to MSC-sTRAIL for cancer therapy (108). Besides, AD-MSCs engineered to express sTRAIL were found to induce apoptotic pathways in pancreatic ductal adenocarcinoma (PDAC) BxPC-3, primary PDAC cells, and MIA PaCa-2 cell lines [109]. On the other hand, sTRAIL secreted by AD-MSCs migrated into the tumour stroma and significantly hampered tumour development in vivo with an antiangiogenic effect and significant reduction in tumour size (109).

MSC-derived extracellular vesicles (EVs) transporting TRAIL are considered a promising anticancer therapeutic approach. EVs derived from MSC-TRAIL were found to exhibit cytotoxicity against neuroblastoma cell line (SHEP-TET), human breast adenocarcinoma line (MDAMB231), renal cancer lines (HA7-RCC and RCC10), malignant pleural mesothelioma lines (H2818, H2810, H2804, and H2795) and lung cancer lines (NCI-H727, NCI-H460 and A549) without any cytotoxic effects towards primary human bronchial epithelial cells (108). Importantly, EVs formed from MSC-sTRAIL induced significant apoptosis in TRAIL-resistant cancer cells, which was enhanced using a CDK9 inhibitor, implying that MSC-derived EVs could be used as an effective antitumour therapy (99).

Similarly, intravenous or intraperitoneal injections of MSC-sTRAIL into a mesothelioma xenograft mouse encouraged tumour shrinkage while reducing local inflammation. These proof-of-concept studies suggest that MSC-sTRAIL may be useful in treating malignant mesothelioma (110). Furthermore, MSC-TRAIL targeting CD133-positive cancer stem cells (CSCs) revealed a potential function of altering apoptosis-related genes in NSCLC (111). Despite their multipotency, MSC-TRAIL exposure to CSCs significantly reduced their proliferation and triggered tumour cell apoptosis in vitro, owing to the activation of the apoptosis intrinsic pathway in CSCs (111). Results of molecular analysis exhibited that altering the expression of harakiri (HRK), DNA damage-inducible alpha (GADD45A), growth arrest, MCL1, BAG cochaperone 3 (BAG3), and NF-κB1 in CSCs was found to be responsible for the antitumour effects of MSC-TRAIL (112).

### Engineered MSCs-Derived Exosomes for Cancer Therapy

#### Inhibiting Tumour Growth

In recent decades, the role of MSCs-Exo on tumour growth has received much attention. According to previous data, miRNA in MSCs-Exo has been linked to the suppression of cancer cell proliferation. Adipose MSCs-Exo is capable of suppressing prostate cancer growth by delivering miRNA-145 to diminish Bcl-xl activity and boost tumour cell apoptosis via the caspase-3 and caspase-7 pathways (113). MiR-302A-loaded UC-MSCs-Exo was reported to suppress the proliferation and migration of endometrial cancer cells by inhibiting the expression of cyclin D1 and the AKT signalling pathway during endometrial cancer treatment (114). Similarly, Wu et al. (115) found that exosomes from UC-MSCs inhibited the development of bladder carcinoma cells by upregulating cleaved caspase-3 and downregulating Akt protein kinase phosphorylation.

Exosome-mediated communication is indispensable for maintaining normal physiological functions. MSCs-Exo can indirectly regulate tumour progression through their effects on signalling pathways. For instance, microRNA-100 carried by MSCs-Exo can inhibit angiogenesis and, consequently, breast cancer progression via the mTOR/HIF1A/VEGF signalling axis (116). miRNAs may have similar tumour-suppressive effects in haematological malignancies. BM-MSCs-Exo directly target the IRF2 gene by secreting miR-222-3p, negatively regulating the IRF2/INPP4B pathway in THP-1 cells, leading to the suppression of leukaemia cell proliferation, the promotion of apoptosis, and the prevention of leukaemia progression (117). Another study investigated the therapeutic role of engineered hUC-MSCs-Exo enriched with miR-302a in endometrial cancer (EC). The authors reported that these exosomes could inhibit EC cell proliferation and migration by suppressing the expression of cyclin D1 and the AKT signalling pathway (114). miRNA-146b derived from MSCs-Exo was shown to attenuate the growth of glioma xenografts in the rat brain. However, the underlying mechanism was not elucidated in the study (118). Similarly, exosomal circ_0030167 derived from BM-MSCs can inhibit the invasion, migration, proliferation, and stemness of pancreatic cancer cells by sponging miR-338-5p and targeting the Wif1/Wnt8/β-catenin axis (119). In general, miRNAs carried by MSCs-Exo can regulate tumour progression, perhaps providing a novel paradigm for future tumour therapy (120).

### Enhancing Drug Sensitivity

Because of their natural intercellular communication function, strong tumour tropism, low immunogenicity, low toxicity, biodegradable characteristics and capability to escape from clearance and cross biological barriers, MSC-derived exosomes have emerged as promising carriers of various biomolecules and chemical agents in cancer treatment. Bioengineered MSC-derived exosomes can encapsulate desired therapeutic cargoes, such as miRNAs, proteins, and drugs. It has been observed that MSCs transfected with synthetic miRNAs can enhance the chemosensitivity of cancer cells by transferring specific miRNAs via exosomes. For example, exosomes derived from miR-199-modified ADMSCs can improve sensitivity to DOX by inhibiting the mTOR signalling pathway in hepatocellular carcinoma in vitro and in vivo [121]. In glioma, miR-199a, a downregulated miRNA in both glioma tissues and cells, has been found to inhibit the proliferation, invasion, and migration of U251 cells in vitro. Furthermore, miR-199a-overexpressing MSC-derived exosomes inhibited glioma progression and enhanced sensitivity to temozolomide (TMZ) by suppressing AGAP2 expression in vitro and in vivo (122). Furthermore, Wharton’s jelly-derived MSC (WJ-MSC)-derived exosomes transfected with miR-124 have been confirmed to sensitise GBM cells to TMZ and inhibit GBM cell proliferation and migration by directly targeting CDK6 in vitro (123). It has been observed that miR-193a expression was downregulated, whereas LRRC1 expression was upregulated in DDP-resistant NSCLC tissues and cells. Meanwhile, BMSC-derived exosomes could inhibit NSCLC progression through upregulating miR-193a and downregulating LRRC1 in vitro and in vivo. Furthermore, BMSC-derived exosomes transfected with miR-193a mimic impaired DDP resistance and inhibited proliferation, migration and invasion by inhibiting LRRC expression in NSCLC (124).

### Inhibiting Metastasis/Invasion

The inhibitory property of MSCs-Exo in metastasis and the premetastatic environment has also been investigated. BMSCs were found to induce dormancy in invasive breast cancer cells by secreting exosomes containing miRNAs (125). Exosomes are made up of multifunctional proteins. MSCs-Exo carries all three immunoproteasome subunits and all seven chains of the 20 S proteasome, implying that exosomes potentially target tumour cells by proteasome transfer (126). MSCs-Exo was also modified to overexpress microRNA-34c-5p (miR-34c) to reveal its effect on the tumour. MSCs-Exo overexpressing miR-34c inhibited the growth of nasopharyngeal carcinoma (NPC) by dampening NPC invasion, migration, proliferation, and epithelial-mesenchymal transition (EMT) process in both in vivo and in vitro experiments, confirming that MSCs-Exo overexpressing miR-34c can be utilised to suppress the progression of NPC by attenuating NPC invasion, migration, proliferation and EMT process (127).

Although MSCs-Exo has a tumour-inhibiting function, research has demonstrated that it promotes tumour growth in time (128). The variances could be due to several factors, including standardised MSC culture conditions, which could impact the overall properties of released bioactive components. Furthermore, the diverse sources of MSC-secreted exosomes are a significant factor. Exosomes produced from BMSCs of patients with multiple myeloma, for example, were able to increase the proliferation of multiple myeloma cells, indicating that other variables, such as high levels of cytokines and adhesion molecules, in addition to elevated miRNA, likely had a role in tumour promotion (129). In contrast, exosomes derived from healthy human BM-MSCs suppressed the growth of multiple myeloma cells by delivering low amounts of miRNA-15a, implying that the origin of MSCs is important for tumour suppression or promotion (130).

### Mesenchymal Stem Cells-Based Cancer Therapies in Clinical Trials

A modest number of registered clinical trials using MSCs to treat solid tumours are currently being conducted. Although some outcomes are yet to be published, many trials were motivated by successful preclinical trials.

#### Gastrointestinal Cancer

The first clinical trial of genetically modified MSCs in humans for gastrointestinal cancers has been published (TREATME1), which uses MSCs to deliver HSV-TK under the control of the CCL5 promoter. The phase I/II clinical trial has been completed (131).

#### Ovarian Cancer

Ovarian cancer was the target of another clinical research employing MSCs. Human MSCs transfected with IFN-β (MSCs-IFNβ) were used in phase I clinical trials sponsored by M.D. Anderson Cancer Centre. The goal of this clinical trial is to determine the maximally tolerated dose of human MSCs-IFNβ that may be given to ovarian cancer patients while also testing its safety (NCT02530047).

#### Anthracycline-Induced Cardiomyopathy (Post-Anticancer Treatment)

SENECA is the first clinical trial to utilise direct cardiac injection of a cell product to treat anthracycline-induced cardiomyopathy, a well-known side effect of anticancer treatment defined by a gradual loss of heart function that eventually leads to dilated cardiomyopathy (DCM). The safety and feasibility of administering all-MSCs transendocardial in a patient population with few therapy options and a poor prognosis will be assessed in this phase I trial. SENECA will inform future larger trials of this new therapeutic technique if the results are promising (132).

#### Lung Cancer

Allogeneic MSCs expressing a full-length form of TRAIL have been employed in a therapeutic strategy for treating lung cancer. The objective of MSCs as a gene therapy vehicle is to deliver TRAIL. TACTICAL is a phase I/II trial evaluating the safety and efficacy of MSC-TRAIL paired with combination therapy in patients with stage IIIB/IV metastatic lung adenocarcinoma. The goal of phase 1 is to determine the recommended phase II dose (RP2D) of MSC-TRAIL when used with pemetrexed/cisplatin chemotherapy. Phase 2 will evaluate the safety and preliminary efficacy of MSC-TRAIL in combination with pemetrexed/cisplatin chemotherapy. TACTICAL is the first clinical trial of this innovative cell and gene therapy, and if the clinical study results are successful, the findings will present an opportunity for allogeneic MSC therapy in cancer in the future (133).

#### Paediatric Tumours

A previous study discussed the findings of a first-in-human, first-in-child trial of Celyvir, an advanced therapeutic drug that combines autologous MSCs with oAd for patients with relapsed solid tumours. Celyvir was created using a bone marrow aspirate and administered intravenously. According to the findings, it is a safe technique for systemically providing repeated doses of oncolytic virotherapy, suggesting further investigation in a phase 2 context. MSCs could be utilised to boost the amount of OVs given to patients while limiting side effects and avoiding direct tumour injections (134).

#### Prostate Cancer

Allogeneic bone marrow-derived MSCs were injected in men with localised prostate cancer in the phase I clinical trial. The main aim of the research was to determine the safety and cancer-homing potential of MSCs. MSCs, however, did not home to primary tumours in sufficient numbers to kill cancer cells or slow tumour growth in this investigation (135).

### Cancer Inducing Potential of Mesenchymal Stem Cells

Many anticancer molecules and agents have been tested as a potential treatment for multiple cancers. However, a major setback is the declining efficacy, resulting in relapse due to a short-half life. Increasing the dosage can overcome the problem but often results in serious side effects such as off-target cytotoxicity (136). This led to the utilisation of MSC as a tool for delivering therapeutic agents in oncology (40). Apart from their endogenous ability to suppress tumour formation and proliferation, MSCs can successfully home to inflamed/tumour sites and express anti-inflammatory abilities. In addition, MSCs are readily available and can be largely reproduced while considerably maintaining their characteristics (40). In the last decade, MSC has been at the forefront of many clinical applications for their relevance in personalised cell-based therapy either as an engineered or non-engineered MSC with remarkable pre-clinical outcomes and promising clinical potential for cancer. Regardless, the therapeutic applications of MSCs are not without challenges. These include the lack of consistency and efficacy of MSCs for MSC-based therapy across research groups (137), which might largely be associated with the heterogeneity in the isolating procedure, donor age and cultivation (expansion and storage protocol) among laboratories, resulting in different non-clonal cultures of MSCs (138).

Other setbacks might be due to the unspecified optimal doses and routes of cell administration (138), the high risk of pro-tumorigenic, immune rejection, and disturbed differentiation capacities. Moreover, differentiating into undesirable tissues that might have a short survival after implantation and their less impressive improvements (139) were also recorded as obstacles. Other detrimental factors include the off-target accumulation and cellular toxicity associated with therapeutic MSCs (139).

There is growing evidence that MSC supports tumour proliferation, differentiation, motility, invasion, and metastasis (140, 141). However, the contributory role of MSC in tumour progression and metastasis is not fully understood, considering the massive potential of MSC. An inventive unravelling of its paracrine secretion and mechanism is vital to understanding the limitation of MSC-based therapy, especially in oncology (138, 141), as many of the clinical values of MSCs were earlier primarily associated with the immune suppressor’s component of the MSCs known as extracellular vesicles made up of exosomes, the multitude of chemokines, and growth factors (142).

The paracrine signalling secretion of MSCs is vital for clinical applications such as treatment for sepsis, graft-versus-host diseases, and diverse autoimmune diseases. These same paracrine components might also support tumorigenesis, motility, and invasiveness in addition to the formation of metastatic niches at potential secondary sites (140) and de novo carcinogenesis (143). Hence, the two-edged sword potential characteristic of MSC in cancer (128). Paracrine secretion of chemokine C-C motif ligand 5 (CCL5), or RANTES, assists MSC migration to the TME. Both the ligand and direct MSC-tumour environment contact interaction enhance the neoplastic properties of tumour cells (140). This subsequently enhances motility, invasion, and metastasis of pre-existing tumours (144).

MSC plays a role in de novo carcinogenesis and cancer development, as evident in their ability to induce EMT, increasing the cancer stem cells (145). A few studies reported that MSC enhanced metastasis through EMT of breast cancer cells (146), lung cancer cells (147) and leukaemia cells (148). Some studies have also shown that MSCs protect cancer cells from ROS-induced apoptosis while inducing aerobic glycolysis (149). This is achieved through the upregulated secretion of stanniocalcin-1 (STC1). The stanniocalcin-1 reduces the intracellular ROS and mitochondrial membrane protein (MMP) while increasing lactate production and accumulation due to reduced pyruvate metabolism (149), shifting the metabolism towards activating higher conserved mammalian uncoupling protein 2 (UPC2) in the microenvironment, and facilitating aerobic glycolysis known as the Warburg effect (150). Other tumour formation and progression enhancers include MSC angiogenic and anti-apoptotic factors, including Bcl-2, Akt, VEGF, HGT, STC1 and IGF (43).

Several studies have shown the possible adverse effects of MSCs on cancer development. Unfortunately, studies exhibiting the correlation between engineered MSCs and tumour development are still scarce. However, due to the ability of MSCs to retain their original phenotype and genetic characteristics post-modification, we speculate that engineered MSCs may also provide cancer-inducing risk in cancer therapy, similar to wild type MSCs (wt-MSCs).

#### Overcoming Cancer Causing Potentials of Mesenchymal Stem Cells

For cell-based clinical applications, MSCs undergo in vitro expansion. Interestingly, while MSCs retain their properties, some cases revealed that culture expansion might lead to the generation and accumulation of cytogenetic. The condition could lead to molecular alterations that could ultimately result in cell malignant transformation (151). Also, the migration ability of MSCs might significantly be reduced or inhibited during culturing. This was reported to be associated with the upregulation of TIMP-3, which inhibits MMP-2, ultimately decreasing trans-endothelial migration (152). As mentioned earlier, the MSC homing mechanism is still not fully understood, and off-targeted migration tend to occur. Evidently, this was reported by previous studies, reporting off-target biodistribution of Odot-labelled hBM-MSCs (153).

Therefore, selecting and preconditioning candidate cells should be the first step to overcome the limitations associated with wt-MSCs and engineered MSCs for cell-based therapy. The donor’s age and health status should be considered, as MSCs from an aged donor have reduced stemness properties. This might deteriorate the disease severity and display senescence-associated secretory phenotype (SASP). Cells derived from aged donors could induce inflammation by recruiting immune cells, contributing to the ageing progress of cells in the lesion microenvironment, and impairing regenerative functions (154).

Selection of MSC with primitive properties, such as young cells derived from by-products at delivery, including umbilical cord blood or WJ, should be used (155). Also important is the need to enhance strategies toward their survival in vivo while maintaining their properties, including stemness, engraftment, and immunomodulatory functions (156). It is also important to strictly adhere to the MSC standard as defined by the ISCT and to perform several quality controls to ensure the safety and efficacy of MSCs for cell therapies (151). Another strategy is to precondition the MSCs by genetic manipulation to enhance the functions, either with cytokines, growth factors, immune receptors or hypoxia. The genetic manipulation preconditioning could also be in the form of co-administration with engineered biomaterials such as a scaffold or tagging the MSCs with drugs (154).

#### Challenges of Mesenchymal Stem Cells-Based Therapy in Clinical Trials

Although experimental and clinical trial applications of MSCs showed promising outcomes in chronic inflammatory and autoimmune diseases, they might still fail to deliver the expected results and are not free from potential adverse events (157). Duijvestein and colleagues (158) reported that at week 6 after MSC administration, three participants had to undergo surgical procedures due to worsening disease. Similarly, 7/12 patients experienced serious adverse events when given a single MSC intravenous infusion (159). However, upon further investigation, exacerbation of the condition was observed in 5/7 participants, while adverse effects in the other 2 participants were probably due to MSC infusions. Locally inoculated allogeneic MSCs in patients suffering from refractory CD and complex fistulas have also been associated with certain adverse reactions like uterine leiomyoma and anal abscess (160–162).

Furthermore, severe adverse events were noticed in moderate to severe UC patients who received multistem therapy comprising nonembryonic tissue and adult bone marrow sources (NCT01240915). These raise concerns about the efficacy and safety of MSC transplants. The ability of MSCs to get engrafted and/or concentrate at the target site, like homing to the mucosa of the intestine and differentiating into epithelial and other cells to promote direct mucosal damage repair, is highly desirable (163). However, relatively few MSCs intravenously administered get engrafted at these target injury sites. Experiments in rodent and dog models have shown that these MSCs get caught up in lung capillaries, during which most are largely cleared, with few going through to the injured target tissue (164). The therapeutic effects produced by MSCs are also known to be short-lived in some studies. Long-term retrospective follow-up investigation expanding a phase II trial indicated recurrence of fistulas in a significant proportion of the study population, with only 7/12 initial responders sustaining complete fistula closure (165, 166).

In addition to the observed adverse events, discrepancies in documented results, and poor migration and engraftment of transplanted MSCs, the therapy is also confronted with unconfirmed long-time adverse events. Again, factors like source, type, and preparation of MSCs, route, quantity, duration, and frequency of administration, as well as other disease and microenvironment factors, need further clarity. Cellular inherent factors and intestinal microenvironment factors that enhance MSC migration, adhesion, proliferation, and cytokine effects need further exploration. MSC modification or engineering techniques and efficiently combined therapeutic approaches should be highlighted to overcome the challenges mentioned.

## Conclusion

Although cancer management has greatly improved in the last couple of years, the efficacy of most treatment options is poor. MSCs and their secreted exosome, in general, have shown huge potential in clinical applications for many ailments, including cancer. Nevertheless, it is also saddled with several shortfalls, especially in tumour therapy. Several engineered MSCs have been designed for cancer treatment and have been extensively investigated in the last few decades. However, the majority did not pass the clinical trials. One of the major clinical challenges is MSCs’ double-edged sword role in tumour therapy. We recommend that this challenge be overcome by performing standard quality controls to test the safety and efficacy of MSCs for cell therapies, including maintaining a standard protocol for selecting, maintaining, storing MSCs, and strictly adhering to the MSC standard as per the ISCT definition.
